# Two-stage comprehensive evaluation of genetic susceptibility of common variants in *FBXO38, AP3B2* and *WHAMM* to severe chronic periodontitis

**DOI:** 10.1038/srep17882

**Published:** 2015-12-08

**Authors:** Dong Shang, Li Dong, Lingfang Zeng, Rui Yang, Jing Xu, Yue Wu, Ran Xu, Hong Tao, Nan Zhang

**Affiliations:** 1 Department of Stomatology, the First Affiliated Hospital, School of Medicine, Xi’an Jiaotong University, Xi’an, China; 2 Department of Respiratory, the First Affiliated Hospital, School of Medicine, Xi’an Jiaotong University, Xi’an, China; 3 Department of Computer Science, Shandong Medical College, Jinan, China; 4 Department of Pediatric Stomatology, Jinan Stomatological Hospital, Jinan, China; 5 Tian-rui Institute of Stomatology, Xi’an, China; 6 Department of Emergency, the First Affiliated Hospital, School of Medicine, Xi’an Jiaotong University, Xi’an, China; 7 Department of Cardiology, the First Affiliated Hospital, School of Medicine, Xi’an Jiaotong University, Xi’an, China

## Abstract

Chronic periodontitis is an oral disorder characterized with gingival inflammation and bone destruction. As the sixth-most prevalent condition affecting more than 743 million people around the world, it is classified as one of the seven destructive oral disorders. Early genetic epidemiological evidence indicated a major role for genetics in periodontal disease development. In this study, we conducted a two-stage comprehensive evaluation of the genetic susceptibility of *FBXO38, AP3B2* and *WHAMM* with the diagnosis of severe chronic periodontitis. A total of 5,065 study subjects from the Han Chinese population consisting of 1,264 cases and 3,801 healthy controls were recruited, and 65 single nucleotide markers related to the three candidate genes were genotyped to investigate the susceptibility of patients with these polymorphisms to severe chronic periodontitis. To increase the coverage of genetic markers, we implemented imputation techniques to extend the number of tested makers to 416. Single marker and haplotype-based analyses were performed, and significant results were obtained for *FBXO38* (rs10043775, *P* = 0.0009) and *AP3B2* (rs11631963-rs11637433, CA, *P* = 9.98 × 10^−5^; rs1864699-rs2099259-rs2278355, ATC, *P* = 3.84 × 10^−8^). Our findings provide direct evidence for the association of *FBXO38* and *AP3B2* with severe chronic periodontitis in the Han Chinese population.

Chronic periodontitis (CP) is a multi-factor complex oral disorder characterized by slowly progressing alveolar bone destruction and attachment loss. A recent systematic review showed that severe CP, as the sixth-most prevalent condition, affected more than 743 million people (10.8%) around the world and that its incidence rate was 701 cases per 100,000 person years^1^. Although CP is usually not painful, it is one of the important risk factors for early tooth loss and imposes a significant health burden to the patients^2^. In addition, numerous studies have shown significant links between periodontal disease and systemic conditions including cardiovascular disease^3^,^4^,^5^, type 2 diabetes mellitus^6^,^7^,^8^, adverse pregnancy outcomes^9^, and osteoporosis^10^,^11^. Therefore, understanding the aetiology and pathology of CP can help to reveal the pathological mechanisms of other major complex disorders and in turn shine light on the development of novel drugs and treatment strategies for these disorders.

Early genetic epidemiological evidence indicated that genetics played a major role in the onset and development of periodontal disease. A twin study of periodontal disease conducted by Corey *et al.* identified significant differences in proband-wise concordance rates between monozygotic (0.38) and dizygotic twins (0.16)^12^. Another twin study has estimated the heritability of adult periodontitis to be approximately 50%^13^. In the past decade, with the development of high throughput genotyping technology, genome-wide association study (GWAS) has been shown to be a powerful tool in identifying susceptibility genes for complex disorder^14^. GWAS of chronic periodontitis have identified several susceptibility genes including *C5AR1*^15^, *DLG2*^15^, *FAM180A*^16^, *MFSD1*^16^, *NCR2*^17^, *NPY*^17^, *EMR1*^17^, *VAV1*^17^, *GLT6D1*^18^, *WHAMM*^19^ and *AP3B2*^19^. However, most of these findings were obtained from samples of European ancestry. Thus, validation of these results in other populations is still necessary.

In our study, we aimed to thoroughly evaluate the genetic risk of three candidate gene polymorphisms (i.e., *FBXO38, AP3B2* and *WHAMM*) on severe CP in the Han Chinese population. All three genes have been shown to be significant (or suggestively significant) susceptibility genes to CP or CP-related symptoms in recent published GWAS papers^19^,^20^ using samples with European ancestry. To investigate the potential susceptibility of these candidate genes, we enrolled 5,065 study subjects of Han Chinese ancestry and genotyped 65 (this number increased by 416 after imputation) pre-selected single nucleotide polymorphism (SNP) markers related to these three genes. A two-stage strategy (discovery and validation stage) was applied in our study, and the potential association signals were tested at both the single SNP and haplotype levels. Additionally, to increase the genomic coverage of SNPs, we also imputed SNPs within two 5-Mb genomic regions that covered the three candidate genes on chromosome 5 and chromosome 15.

## Materials and Methods

### Subjects

The subjects involved in this study included 5,065 individuals ranging from 28–75 years of age. Among them, there were 1,264 cases and 3,801 healthy control subjects. All of the study subjects were enrolled from October 2010 to August 2014. Severe chronic periodontitis patients were recruited from the inpatient and outpatient clinical services at the First Affiliated Hospital of Xi’an Jiaotong University and the Stomatology Hospital of the Fourth Military Medical University. Severe chronic periodontitis was defined as a patient who presented at least two interproximal sites with clinical attachment loss (CA loss) ≥ 6 mm, not in the same tooth, and probing depth (PD) ≥ 5 mm in one or more interproximal sites, excluding third molars. Three examiners in our study were trained and calibrated by the author N. Z. in accordance with aforementioned diagnosis criteria. All four examiners are dentists. They performed clinical examinations by using a manual probe (UNC-15, Hu-Friedy Manufacturing Company, Inc., Chicago, IL, USA), and all were calibrated on 40 chronic periodontitis patients probed twice before conducting periodontal examinations in our study. In order to facilitate the interpretation of measurement reliability, we reported kappa statistics and inter/intra-class correlation coefficients for PD. The Kappa index and intra-class correlation coefficient for PD ranged from 0.77 to 0.92 and 0.73 to 0.87 for intra-examiner calibration. Inter-examiner Kappa index and intra-class correlation coefficient of agreement in measurements varied from 0.81 to 0.91 and 0.77 to 0.86 for PD (Supplemental Table S1). These results evidenced a high degree of consistency in the measurements and ensured the validity of subsequent periodontal examinations.

The healthy control subjects were enrolled when there were no signs of any periodontal disease at the time of sample collection and no history of periodontal disease. All individuals enrolled in this study were non-smokers. All of our subjects were unrelated Han Chinese residing in Shaanxi Province from similar socio-economic levels. Participants who reported the following characteristics were excluded from the study: use of orthodontic appliances, chronic anti-inflammatory drugs or immunosuppressive chemotherapy, antibiotics within the previous 3 months, chronic inflammatory diseases, a history of diabetes mellitus, hepatitis, HIV infection, nephritis, bleeding or autoimmune disorders, diseases with severe commitment of immune function, current pregnancy or breastfeeding, or received periodontal therapy in the preceding 6 months. The peripheral blood sample was drawn from a vein into a sterile tube containing ethylenediaminetetraacetic acid (EDTA). The plasma samples were stored at −80 °C.

The research protocol was approved by the Ethical Committee of Xi’an Jiaotong University. We obtained written informed consent from all participants after the objectives and procedures of this study were fully explained. All of the experiments on humans were performed in accordance with the ethical guidelines of the Declaration of Helsinki (version 2002).

### SNP selection and genotyping

In this project, we implemented a two-stage experimental cost saving strategy including 1) a discovery stage in which we genotyped a relatively larger set of markers in a smaller subset of subjects (1,783 subjects consisting of 471 cases and 1,312 controls) and 2) a validation stage in which we only genotyped a smaller set of markers that passed the screening P value threshold (0.05) in a relatively larger set of subjects (3,282 subjects with 793 cases and 2,489 controls). Three candidate genes from two genomic regions including *FBXO38, AP3B2* and *WHAMM* were selected for this study. All of the SNP markers located within ±10 kb of the gene regions and with minor allele frequency (MAF) greater or equal to 0.05 in the Hapmap Han Chinese data^21^ were selected. In addition, another set of SNPs related to these genes that were reported to be significant in recent GWAS papers was also selected. The total number of SNPs genotyped in the discovery stage was 65.

Genomic DNA was isolated from the peripheral blood leukocytes according to the manufacturer’s protocol (Genomic DNA kit, Axygen Scientific Inc., CA, USA). The samples of patients and controls were mixed on the same plates, and a double-blind procedure was performed. The DNA was stored at −80 °C for genotyping. The genotyping was conducted for all of the SNPs using the Sequenom MassARRAY matrix-assisted laser desorption ionization-time of the flight mass spectrometry platform (Sequenom, San Diego, CA, USA) on the genomic DNA isolated from the peripheral leukocytes. Final data were released using Typer Analyzer software (Sequenom).

### Statistical analysis

We conducted a comprehensive power analysis with Genetic Power Calculator^22^. In the power analysis, we calculated the statistical power by setting different relative risk (1.1–1.5) and MAF (0.05, 0.1, 0.2, 0.3, 0.4) of the underlying risk allele. We set the SNP marker allele to be 0.287 (average MAF of the 65 SNPs genotyped in the screening stage). Results of the statistical power analysis were summarized in Supplemental Table S2. As shown in the Supplemental Table S2, around 80% statistical power could be achieved if the underlying risk allele has a relative risk of 1.2–1.3. We utilized Polyphen2^23^ to predict the potential functional effects of significant non-synonymous SNPs. The statistical computing software R^24^ was utilized for analysis of general purpose. We implemented a logistic model using genetic analysis software Plink^25^ to investigate the association between genetic polymorphisms and disorder status in both the discovery and validation stage. Bonferroni correction was applied in the validation stage to address the multiple comparison problem. Gender and age were included in the logistic model as a covariate. Plink was also utilized to test for Hardy-Weinberg equilibrium (HWE) in control samples and to calculate the minor allele frequency (MAF) for each marker in both stages. In addition, the haplotype frequency estimation and haplotype-based association analysis were also conducted by Plink. We constructed the LD plot using Haploview^26^ based on the data in the discovery stage. Imputation based on our discovery stage data was implemented using IMPUTE2 software^27^ with HapMap phase III CHB+JPT+CHD data as a reference panel. Association tests based on the imputed dosage data were conducted with the software SNPTEST v2^28^. We utilized the parameter of “average certainty” calculated in IMPUTE2 as the main indicator of imputation quality. The threshold of this indicator was chosen by exploring the patterns of Q-Q plots based on the P-values of results of association analysis based on multiple marker sets obtained using different certainty thresholds.

## Results

**Figure 1:**
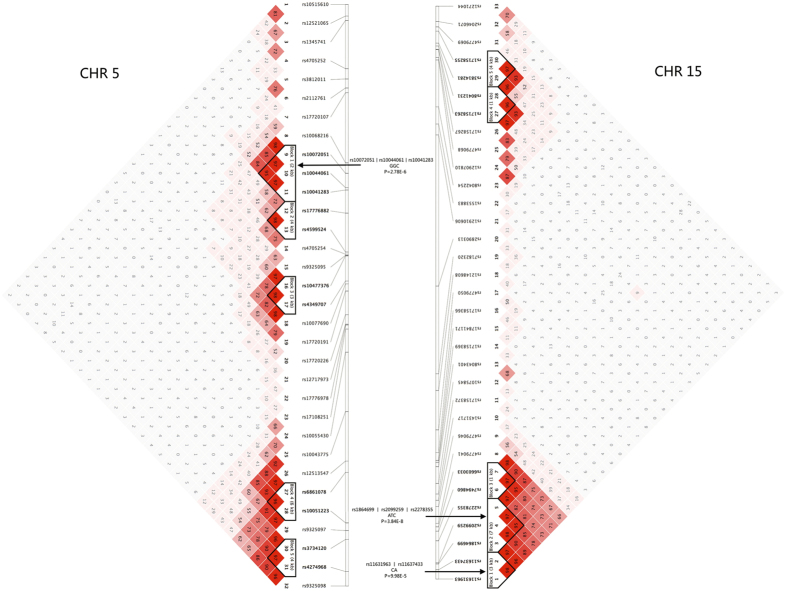
LD structure and significantly associated haplotypes based on the discovery stage data. Ten LD blocks was indicated by the shaded matrices, and three significant haplotypes were identified in the relevant LD blocks indicated by arrows.

The characteristics of these study subjects were summarized in Table 1. Seven SNPs passed the discovery stage screening threshold (P < 0.05) and were sent for genotyping in the validation stage. The results of single SNP-based association analysis of these 7 SNPs in both stages were summarized in Table 2. The full association results of discovery stage could be found in Supplemental Table S3. As presented in Table 2, only 1 SNP, rs10043775 (*P* = 0.0009), passed the Bonferroni corrected P-value threshold (0.05/7 ≈ 0.007) in the validation stage. Ten linkage disequilibrium (LD) blocks were identified on the two candidate genomic regions. Haplotype association analysis was performed based on the data from the discovery stage. The LD blocks and significant findings of haplotype analysis based on the discovery stage genotype data were shown in Fig. 1. As suggested in Fig. 1, three significant haplotypes were successfully identified. One significant haplotype covered part of the *FBOX32* gene region (rs10072051-rs10044061-rs10041283, GGC, *P* = 2.78 × 10^−6^), and the other two (rs11631963-rs11637433, CA, *P* = 9.98 × 10^−5^; rs1864699-rs2099259-rs2278355, ATC, *P* = 3.84 × 10^−8^) covered part of the *AP3B2* gene region. The full results of haplotype analysis were summarized in Supplemental Table S4.

**Figure 2:**
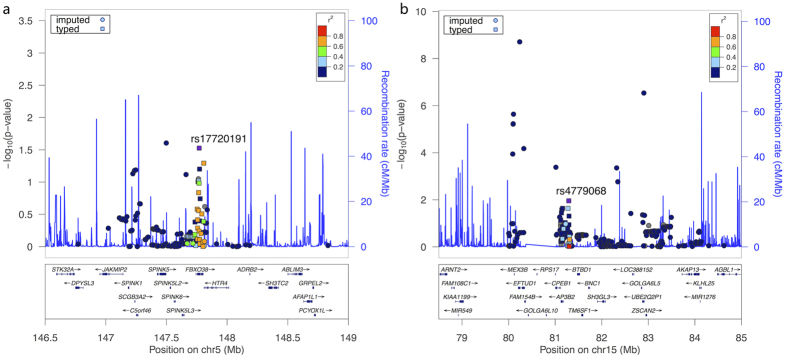
Regional association plots based on imputed regions on chromosome 5 (a) and chromosome 15 (b). Imputed SNPs were indicated as circle and genotyped SNPs were indicated as square. The most significant genotyped SNPs were chosen as reference SNPs in both plots (rs17720191 and rs4779068).

The imputation was implemented for two 5-Mb genomic regions including a 1.46 × 10^8^–1.51 × 10^8^ region on chromosome 5 (including *FBXO38*) and a 8.0 × 10^7^–8.5 × 10^7^ region on chromosome 15 (including *AP3B2* and *WHAMM*). We chose 0.8 as the average certainty threshold to exclude those potential imputed SNPs with low accuracy. This threshold was chosen by exploring the patterns of Q-Q plots based on the P-values of association analysis based on multiple marker sets obtained by using different certainty thresholds (Supplemental Figure S1). In addition, we only focused on the common SNPs, so we also applied MAF ≥ 0.01 as another filter criterion. After applying these filters, 416 SNPs were successfully imputed and tested for association based on the data of 65 genotyped SNPs in the discovery stage. We summarized the imputed SNPs with nominal significance (*P* < 0.05) from association analysis in Supplemental Table S5, and the visualization of the results of association tests based on the two imputed genomic regions were shown in Fig. 2 (using the most significant genotyped SNP as a reference SNP). The most significant SNPs within these regions were two imputed SNPs: rs4362936 (chr5, *P* = 0.0248) and rs11636500 (chr15, *P* = 1.95 × 10^−9^). In addition, we also explored the regional association pattern of these two imputed regions using the most significant imputed SNPs as reference SNPs, and the results were shown in Supplemental Figs S2 and S3.

## Discussion

In a large-scale candidate gene-based genetic association study of severe CP from a Han Chinese population, we obtained several significant association signals. The SNP rs10043775, which is in the exonic region of *FBXO38*, passed the discovery stage screening (*P* = 0.0121) and was further confirmed in the validation stage (*P* = 0.0009). The C allele carriers of this SNP increased the odds of suffering from severe CP by 24.1% compared to the reference allele carriers. Rs10043775 is a missense variant that causes a Serine to Proline change; however, according to the analysis of Polyphen2, this change may not cause any damage to the encoded protein. In a recently published GWAS study based on samples of European ancestry, rs10043775 was found to be suggestively associated with periodontal pathogen colonization^20^. The phenotype utilized in this study was pathogen colonization but not CP diagnosis. In this sense, our finding successfully established a direct link between rs10043775 (and the gene *FBXO38*) and severe CP with Han Chinese population samples. The statistical power of this GWAS study was more or less limited by its relatively small sample size (n = 1,020) and huge number of tests, and therefore, only “suggestive” results were obtained. In our study, we performed a candidate gene-based study and recruited far more samples (n = 5,065) to guarantee the sufficient statistical power. In addition to the single marker-based analysis, haplotype analysis produced more significant findings. Among the three significant findings in haplotype analysis, the significant haplotype rs10072051-rs10044061-rs10041283 (GGC, *P* = 2.78 × 10^−6^), which covered the *FBXO38* gene region, was a replication of the results from single marker-based analysis. The two other significant haplotypes, rs11631963-rs11637433 (CA, *P* = 9.98 × 10^−5^) and rs1864699-rs2099259-rs2278355 (ATC, *P* = 3.84 × 10^−8^), covered part of the *AP3B2* gene region. This gene was previously identified as a significant susceptibility gene for CP using samples of European ancestry^19^. In addition to these significant findings, we failed to detect any significant signals for WHAMM. Neither single marker nor haplotype-based association analysis resulted in any positive findings for this gene despite it being reported to be a significant candidate gene for CP in a previous GWAS study^19^. There may be several reasons for this inconsistent result with the previous study. One is that CP, as a complex disorder, can be associated with multiple genes due to genetic heterogeneity. The difference in ethnic ancestry of the study samples (Han Chinese vs. European) may also affect the results of the association test. Moreover, the statistical method used for the association test in the GWAS study was a gene-centric method, which is different from single marker-based association tests. The significant signal for *WHAMM* identified with this method is due to the contributions of multiple common SNPs within the genetic region, and it is difficult to replicate this result by conducting single marker-based association studies with only a few genotyped SNPs.

Genotype imputation is a widely used statistical technique to predict or impute genotypes that are not directly assayed in a sample of individuals^28^. Simulations have shown that imputation can lead to a boost in the power of GWAS^29^. For candidate gene-based association studies, imputed SNPs may sometimes show larger associations and imputation can be used as a powerful tool for fine-mapping those untyped variants when funding is limited^30^. Through this technique, we successfully imputed and tested 416 SNPs, which increased our marker set by approximately 8 times (416 vs. 65). The strict filtering criteria (certainty >0.8 and MAF >0.01) we implemented ensured that we could exclude most if not all of the inaccurately imputed markers. The information from these imputed SNPs offered us a new way to scrutinize our genetic data. The most significant SNPs for both regions (chr5 and chr15) were imputed but not genotyped SNPs. However, when carefully checking the regional association plots made using these top significant SNPs as reference SNPs (Supplemental Figs S2 and S3), we failed to identify a clustering pattern, which might indicate that these top hits might be spurious.

Without further evidence based on functional studies in animal models, it is too early to determine the pathological significance of these susceptibility genes and severe CP. However, it is still meaningful to have a general discussion of the biological functions of these genes. F-box only protein 38, a protein encoded by the gene *FBXO38* (5q32), is a transcriptional co-activator of Krueppel-like factor 7 (Klf7), and Klf7 has long been related to neuronal development^31^. Previous studies have linked *FBXO38* to distal spinal muscular atrophy^31^ and cancer development and progression^32^. *WHAMM* (15q25.2) encodes a protein that regulates the membrane dynamics and functions at the interface of microtubule and actin cytoskeletons^33^. It is related to the process of actin nucleation, mediating Golgi membrane association and microtubule binding^34^. Adaptor protein-3, beta-2 subunit is a protein encoded by *AP3B2* (15q25.2). As a subunit of Adaptor protein-3 (AP3), it helps to constitute this heterotetrameric vesicle-coat protein complex, which is believed to play an important role in neuron-specific functions^35^. Early mouse studies have shown that overexpression of the neuronal AP3 subunit, including Ap3b2, can produce a large number of small volume vesicles that release smaller amounts of neurotransmitters compared to mice with AP3 deletions^34^. AP3, as a protein complex related to neuron functions, has been found to be related to some psychiatric disorders such as schizophrenia^35^. Despite all of these data, the underlying mechanisms that link these neuronal functions and membrane dynamics to severe CP are still unknown and more research would be necessary.

As a common SNP-based candidate gene association study, our study suffers from several limitations. First, by targeting a relatively small set of common SNPs, we dropped all of the rare variants within these three gene regions. Despite the limitation of statistical analysis methods for rare variant-based association analysis^36^, recent genetic epidemiology studies have indicated that the rare variants have played an important role in the onset and development of complex disorders^37^,^38^,^39^,^40^,^41^. Second, candidate gene-based association study designs only focus on a few potential susceptibility genes but ignore the fact that the biological mechanisms behind most of the complex traits or disorders always involve multiple genes and that these genes interact with each other in a systematic manner^42^,^43^,^44^,^45^,^46^. Single gene-based analysis without incorporating knowledge of biological pathways may only offer us limited knowledge about CP^47^,^48^. In addition, we obtained the smoking status of study subjects solely based on self-report and its validity might be questionable.

## Conclusion

In summary, our study successfully identified two candidate genes, *FBXO38* and *AP3B2*, to be significantly associated with the diagnosis of severe CP in Han Chinese population samples. In the future, further studies are still needed to validate our findings in other cohorts and populations. Additionally, a targeted or exome sequencing study design and pathway-based analysis may provide more evidence to reveal the genetic predisposition of severe CP.

## Additional Information

**How to cite this article**: Shang, D. *et al.* Two-stage comprehensive evaluation of genetic susceptibility of common variants in *FBXO38, AP3B2* and *WHAMM* to severe chronic periodontitis. *Sci. Rep.* **5**, 17882; doi: 10.1038/srep17882 (2015).

## Supplementary Material

Supplementary Information

## Figures and Tables

**Table 1:** Characteristic information of study subjects.

	**Stage 1 (discovery)**	**Stage 2 (validation)**	**Total**	
Gender				
Male (%)	896 (50)	1711(45)	2607 (51)	
Female (%)	887 (50)	1571(55)	2458 (49)	
Status				
Case (%)	471 (26)	793 (21)	1264 (25)	
Control (%)	1312 (74)	2489 (79)	3801 (75)	
Age				
Mean (SD.)	48.83 (13.12)	48.76 (13.05)	48.78 (13.08)	
Range (years)	28–74	28–75	28–75	

SD means standard deviation.

**Table 2:** Results of association analysis based on single SNP that passed the *p* value threshold of discovery stage.

**SNP**	**CHR**	**POS**	**Gene**	**Stage 1 (discovery)**						**Stage 2 (validation)**					
				**HWE**	**MAF_A**	**MAF_U**	**A1**	***P***	**OR**	**HWE**	**MAF_A**	**MAF_U**	**A1**	***P***	**OR**
**rs10043775**	**5**	**148425557**	***FBXO38***	0.8736	**0.2866**	**0.2435**	**G**	**0.0121**	**1.24**	0.9107	**0.2844**	**0.2431**	**G**	**0.0009**	**1.24**
rs10477376	5	148405651	*FBXO38*	0.5725	0.2484	0.2130	G	0.0248	1.23	0.4591	0.2339	0.2053	G	0.0164	1.18
rs17108251	5	148415019	*FBXO38*	0.6213	0.2378	0.2024	G	0.0266	1.22	0.4610	0.2377	0.2188	G	0.1138	1.11
rs10044061	5	148396934	*FBXO38*	0.6698	0.2548	0.2195	T	0.0270	1.22	0.8603	0.2610	0.2280	T	0.0073	1.19
rs10068216	5	148393755	*FBXO38*	0.7643	0.2569	0.2233	C	0.0379	1.20	1.0000	0.2509	0.2328	C	0.1395	1.10
rs9325097	5	148440782	*FBXO38*	0.3198	0.2420	0.2111	A	0.0496	1.19	0.4959	0.2503	0.2292	A	0.0847	1.12
rs2890313	15	82748701	*AP3B2/FSD2*	0.9108	0.1741	0.1441	C	0.0303	1.24	0.8163	0.1627	0.1529	C	0.3451	1.08

The Significant association signal was indicated in bold. The *P* value thresholds used in discovery stage and validation stage are 0.05 and 0.007 (0.05/7), respectively. MAF_A and MAF_U stand for the MAF of cases and controls, respectively.
