# Supplementary material for: Two-stage comprehensive evaluation of genetic susceptibility of common variants in FBXO38, AP3B2 and WHAMM to severe chronic periodontitis
Source: Sci Rep. 2015 Dec 8;5:17882. doi: 10.1038/srep17882 (PMC4672326; doi:10.1038/srep17882)
Supplement: Supplementary Information [file srep17882-s1.pdf]

Title: Two-stage comprehensive evaluation of genetic susceptibility of common variants in *FBXO38, AP3B2* and *WHAMM* to severe chronic periodontitis

Author names and affiliations: Dong Shang [a,b,*], Li Dong [c,*], Lingfang Zeng [d], Rui Yang [e], Jing Xu [f], Yue Wu [g], Ran Xu [a] , Hong Tao [a] and Nan Zhang [a]

[a] Department of Stomatology, the First Affiliated Hospital, School of Medicine, Xi'an Jiaotong University, Xi'an, China

[b] Department of Respiratory, the First Affiliated Hospital, School of Medicine, Xi'an Jiaotong University, Xi'an, China;

[c] Department of Computer Science, Shandong Medical College, Jinan, China;

[d] Department of Pediatric Stomatology, Jinan Stomatological Hospital, Jinan, China;

[e] Tian-rui Institute of Stomatology, Xi'an, China;

[f] Department of Emergency, the First Affiliated Hospital, School of Medicine, Xi'an Jiaotong University, Xi'an, China

[g] Department of Cardiology, the First Affiliated Hospital, School of Medicine, Xi'an Jiaotong University, Xi'an, China

* These authors contributed equally to this work.

Corresponding Author:

Hong Tao, Ph.D., M.D., Department of Stomatology, the First Affiliated Hospital, School of Medicine, Xi'an Jiaotong University, 277 Yanta West Road, Xi'an, China, 710061. Tel: +86-29-85324154, Fax: +86-29-85324142, E-mail: taohong0228@126.com

Nan Zhang, Ph.D., M.D., Department of Stomatology, the First Affiliated Hospital, School of Medicine, Xi'an Jiaotong University, 277 Yanta West Road, Xi'an, China, 710061. Tel: +86-29-85324156, Fax: +86-29-85324141, E-mail: nanzhangnz@126.com

Table S1 Results of intra- and inter-examiner calibration for pocket depth

Trainee	Intra-examiner calibration		Inter-examiner calibration					
	K-test	ICC	K-test	ICC	K-test	ICC	K-test	ICC
1	0.84	0.81	2: 0.81	2: 0.77	3: 0.91	3: 0.81	4: 0.83	4: 0.80
2	0.77	0.73	3: 0.90	3: 0.83	4: 0.82	4: 0.79		
3	0.80	0.76	4: 0.91	4: 0.86				
4	0.92	0.87						

K-test = Kappa test; ICC = Intra-class Coefficient Correlation

Table S2. Statistical power of single SNP based analysis.

Relative risk	MAF of the underlying risk allele				
	0.05	0.1	0.2	0.3	0.4
1.1	0.0598	0.0863	0.1778	0.2696	0.1594
1.2	0.0901	0.2038	0.5512	0.7682	0.4701
1.3	0.1431	0.4007	0.8759	0.9770	0.7844
1.4	0.2203	0.6267	0.9834	0.9992	0.9422
1.5	0.3196	0.8122	0.9989	1	0.9889

Table S3. Results of single marker based association analysis in discovery stage.

SNP	CHR	POS	Gene	HWE	MAF	A1	P	OR
5	rs10515610	148373526	FBXO38	0.7779	0.2743	G	0.1070	1.15
5	rs12521065	148374991	FBXO38	0.6468	0.2448	T	0.0720	1.17
5	rs1345741	148375556	FBXO38	0.9511	0.3486	C	0.1323	1.13
5	rs4705252	148381722	FBXO38	0.6893	0.2956	C	0.6012	1.05
5	rs3812011	148383034	FBXO38	0.9411	0.2493	T	0.8164	1.02
5	rs2112761	148384188	FBXO38	0.535	0.339	T	0.3030	1.09
5	rs17720107	148384797	FBXO38	0.6536	0.2468	G	0.5149	1.06
**5**	**rs10068216**	**148393755**	**FBXO38**	**0.8736**	**0.2322**	**G**	**0.0379**	**1.20**
5	rs10072051	148395099	FBXO38	0.372	0.251	G	0.2706	1.10
**5**	**rs10044061**	**148396934**	**FBXO38**	**0.5725**	**0.2288**	**G**	**0.0270**	**1.22**
5	rs10041283	148397235	FBXO38	0.6022	0.2005	G	0.5374	1.06
5	rs17776882	148400189	FBXO38	0.6248	0.3472	A	0.4350	1.06
5	rs4599524	148405164	FBXO38	0.7501	0.3231	C	0.1567	1.12
5	rs4705254	148405446	FBXO38	0.536	0.4394	C	0.1498	1.12
5	rs9325095	148405595	FBXO38	0.1493	0.2616	G	0.4736	1.06
**5**	**rs10477376**	**148405651**	**FBXO38**	**0.6213**	**0.2224**	**G**	**0.0248**	**1.23**
5	rs4349707	148408875	FBXO38	1	0.2796	C	0.5839	1.05
5	rs10077690	148409235	FBXO38	0.6482	0.244	G	0.1619	1.13
5	rs17720191	148410158	FBXO38	1	0.3231	G	0.1283	1.13
5	rs17720226	148413176	FBXO38	0.3574	0.1447	A	0.0848	1.20
5	rs12717973	148414408	FBXO38	0.7101	0.2468	A	0.8754	1.01
5	rs17776978	148414498	FBXO38	0.3473	0.1405	T	0.1734	1.15
**5**	**rs17108251**	**148415019**	**FBXO38**	**0.6698**	**0.2117**	**T**	**0.0266**	**1.22**
5	rs10055430	148417976	FBXO38	0.8525	0.3292	A	0.2691	0.91
**5**	**rs10043775**	**148425557**	**FBXO38**	**0.7643**	**0.2549**	**C**	**0.0121**	**1.24**
5	rs12513547	148431902	FBXO38	0.7712	0.2616	C	0.1215	1.14
5	rs6861078	148433338	FBXO38	0.6163	0.3295	T	0.7291	1.03
5	rs10051223	148439803	FBXO38	0.8525	0.3345	T	0.9217	0.99
**5**	**rs9325097**	**148440782**	**FBXO38**	**0.3198**	**0.2193**	**A**	**0.0496**	**1.19**
5	rs3734120	148441906	FBXO38	0.05581	0.2496	T	0.8926	0.99
5	rs4274968	148446027	FBXO38	0.1766	0.2426	T	0.9926	1.00
5	rs9325098	148447875	FBXO38	0.2857	0.2672	T	0.3500	1.08
15	rs11631963	82649451	AP3B2	0.652	0.4248	T	0.4637	0.95
15	rs11637433	82652585	AP3B2	0.7367	0.4403	A	0.6968	1.03
15	rs1864699	82654567	AP3B2	0.5053	0.4627	A	0.6677	1.03
15	rs2099259	82654937	AP3B2	0.7383	0.4487	C	0.4005	0.94
15	rs2278355	82662403	AP3B2	0.8202	0.4148	T	0.9390	0.99
15	rs7494860	82665667	AP3B2	0.867	0.4481	T	0.9751	1.00
15	rs6603033	82667123	AP3B2	0.8243	0.4635	A	0.8267	1.02
15	rs4779041	82668307	AP3B2	0.8231	0.4462	G	0.7908	1.02
15	rs4779046	82674020	AP3B2	0.7128	0.2549	A	0.2507	1.10
15	rs1431717	82676117	AP3B2	0.9408	0.2482	C	0.7184	1.03
15	rs17158372	82678246	AP3B2	0.1771	0.1052	G	0.3891	1.11

15	rs1075845	82681865	AP3B2	1	0.2785	T	0.4780	1.06
15	rs8043401	82687507	AP3B2	0.4315	0.4428	A	0.0919	1.14
15	rs17158369	82688976	AP3B2	0.4245	0.1408	T	0.9201	1.01
15	rs17841171	82689636	AP3B2	0.2314	0.2291	G	0.0953	1.16
15	rs17158366	82692328	AP3B2	0.6411	0.2372	A	0.1518	1.13
15	rs4779050	82699986	AP3B2	0.7395	0.4762	T	0.2711	1.09
15	rs12148608	82713350	AP3B2	0.4199	0.2445	A	0.1176	0.87
15	rs7182320	82719000	AP3B2	1	0.2574	A	0.5639	1.05
15	**rs2890313**	**82748701**	**AP3B2/FSD2**	**0.9108**	**0.152**	**C**	**0.0303**	**1.24**
15	rs12910606	82799903	WHAMM	0.5006	0.2515	A	0.0611	1.18
15	rs1553883	82803597	WHAMM	0.6323	0.1739	A	0.7963	0.97
15	rs8042254	82809417	WHAMM	0.5325	0.2779	A	0.4005	1.07
15	rs12907810	82825299	WHAMM	0.8778	0.2339	C	0.8808	0.99
15	rs4779068	82826149	WHAMM	0.9017	0.3298	A	0.1768	0.90
15	rs17158267	82828837	WHAMM	0.8425	0.168	A	0.6235	1.05
15	rs17158263	82828908	WHAMM	0.9429	0.2644	T	0.2966	1.09
15	rs8041231	82830043	WHAMM	0.9087	0.4069	T	0.6178	1.04
15	rs3814281	82831014	WHAMM	0.9373	0.2271	A	0.9240	1.01
15	rs17158255	82835068	WHAMM	0.8543	0.1848	A	0.8434	1.02
15	rs4779069	82836510	WHAMM	0.1375	0.3357	C	0.9675	1.00
15	rs2046071	82838299	WHAMM	0.946	0.281	A	0.4279	0.93
15	rs1271044	82843501	WHAMM	0.3812	0.3374	G	0.0894	1.17

SNPs that passed the discovery stage threshold were shown in bold.

Table S4. Results of haplotype association analysis based on the discovery stage data.

Locus	CHR	Haplotype	Haplotype freq.	Case / control frequencies	Chi square	*P*-value
H1	5					
		TAC	0.741	0.718, 0.749	3.293	0.0696
		GGG	0.194	0.192, 0.195	0.03	0.8632
		GGC	0.03	0.052, 0.022	21.961	**2.78×10^{-6}**
		GAC	0.027	0.020, 0.030	2.365	0.1241
H2	5					
		GA	0.649	0.636, 0.654	1.038	0.3083
		AC	0.32	0.333, 0.315	1.096	0.2951
		AA	0.028	0.023, 0.029	0.812	0.3674
H3	5					
		AT	0.717	0.707, 0.721	0.713	0.3986
		GC	0.219	0.242, 0.211	3.766	0.0523
		AC	0.06	0.045, 0.066	5.372	0.0205
H4	5					
		GC	0.659	0.651, 0.662	0.362	0.5474
		TT	0.323	0.318, 0.324	0.108	0.7424
		GT	0.012	0.015, 0.011	1.036	0.3087
H5	5					
		CG	0.746	0.744, 0.746	0.015	0.9027
		TT	0.238	0.233, 0.239	0.128	0.7208
		TG	0.012	0.014, 0.011	0.446	0.5042
H6	15					
		CG	0.556	0.551, 0.558	0.136	0.7118
		TA	0.421	0.412, 0.424	0.455	0.4999
		CA	0.019	0.034, 0.014	15.141	**9.98×10^{-5}**
H7	15					
		GTC	0.528	0.512, 0.534	1.248	0.264
		ACT	0.406	0.396, 0.409	0.533	0.4652
		ACC	0.039	0.034, 0.040	0.692	0.4054
		ATC	0.019	0.039, 0.011	30.229	**3.84×10^{-8}**
H8	15					
		CC	0.531	0.523, 0.533	0.288	0.5915
		TA	0.442	0.439, 0.443	0.047	0.828
		CA	0.021	0.028, 0.019	2.663	0.1027
H9	15					
		CG	0.587	0.576, 0.591	0.677	0.4105
		TT	0.258	0.267, 0.255	0.473	0.4914
		CT	0.149	0.147, 0.149	0.017	0.8958
H10	15					
		GG	0.769	0.762, 0.772	0.353	0.5525
		AA	0.181	0.177, 0.182	0.129	0.7192
		AG	0.046	0.051, 0.044	0.716	0.3976

Significant *P*-values were shown in bold.

Table S5. The association test results of imputed SNPs with nominal significance.

SNP	CHR	POS	alleleA	alleleB	MAF	P	Function	Gene
rs11636500	15	82158686	A	C	0.0297	1.95E-09	intron	*EFTUD1*
rs1849287	15	84559840	C	G	0.0365	2.91E-07	intron	*UBE2Q2P1*
rs11630156	15	82025385	A	G	0.0717	2.31E-06	unknown	—
rs17267218	15	82018946	A	C	0.0703	5.90E-06	unknown	—
rs9788775	15	82018008	A	C	0.0703	6.10E-06	unknown	—
rs7183805	15	82247090	A	G	0.0512	6.67E-05	intron	*EFTUD1*
rs7162970	15	82009591	C	T	0.0982	0.0001	unknown	—
rs2277566	15	82549415	C	T	0.0132	0.0004	intron	*CPEB1*
rs7167818	15	83860332	C	T	0.0398	0.0004	intron	*ADAMTSL3*
rs10220741	15	83884806	A	G	0.0401	0.0017	intron	*ADAMTSL3*
rs12910606	15	82799903	A	C	0.1820	0.0068	intron	*FSD2*
rs8041231	15	82830043	G	T	0.3784	0.0084	intron	*WHAMM*
rs4362936	5	148138826	C	T	0.1009	0.0248	unknown	—
rs12901468	15	84164180	A	G	0.0228	0.0381	unknown	—
rs150968	15	84532635	G	T	0.0237	0.0436	intron	*UBE2Q2P1*
rs2341203	15	84562455	C	T	0.0238	0.0455	intron	*UBE2Q2P1*
rs2879971	15	84574237	C	T	0.0238	0.0460	intron	*LINC00933*

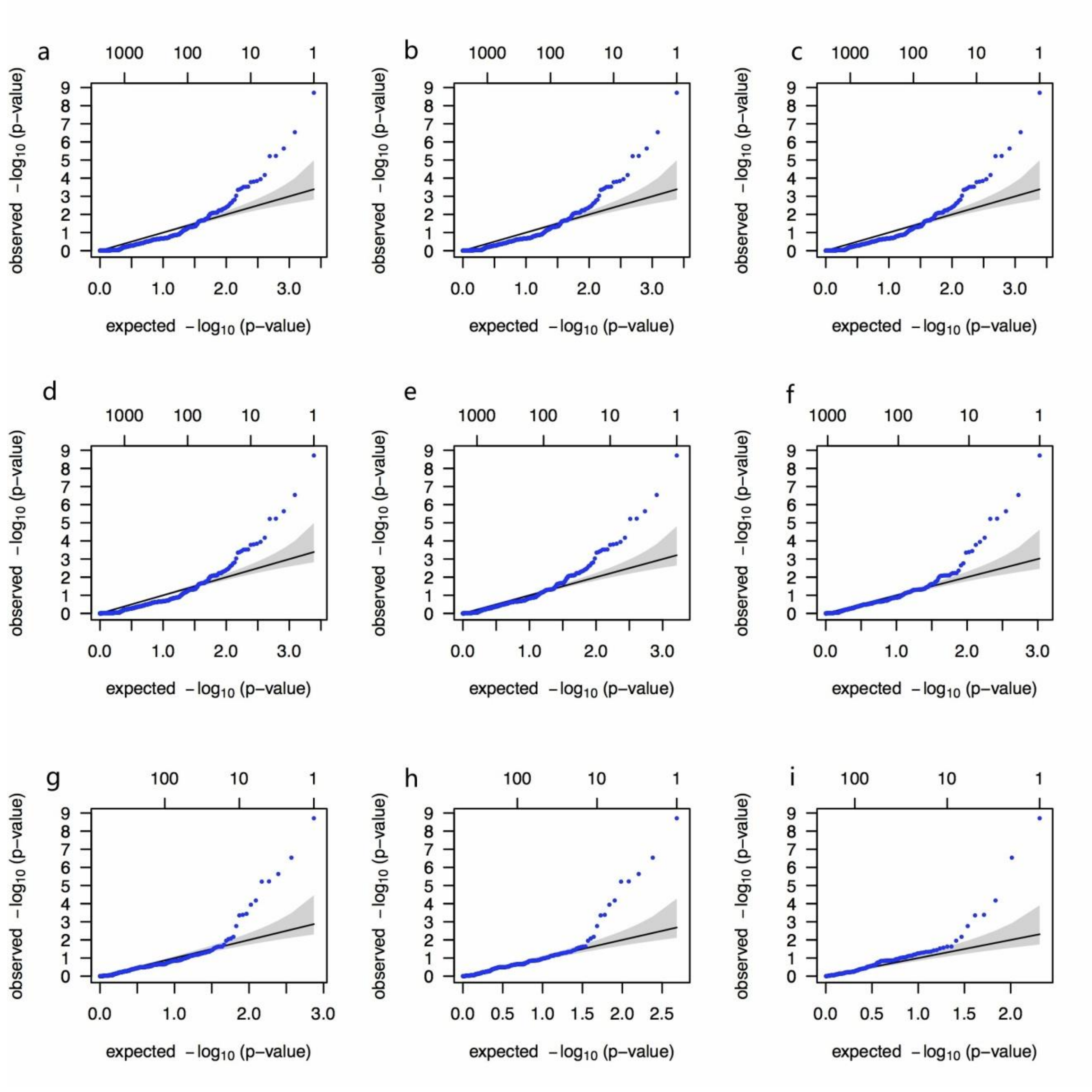

Figure S1. **The Q-Q plots made by association test results based on marker set filter by different certainty thresholds.** The certainty thresholds used in a, b, c, d, e, f, g, h, i are 0.1, 0.2, 0.3, 0.4, 0.5, 0.6, 0.7, 0.8, 0.9 respectively. No significant deviance from expected line could be found when certainty threshold was chosen as 0.8.

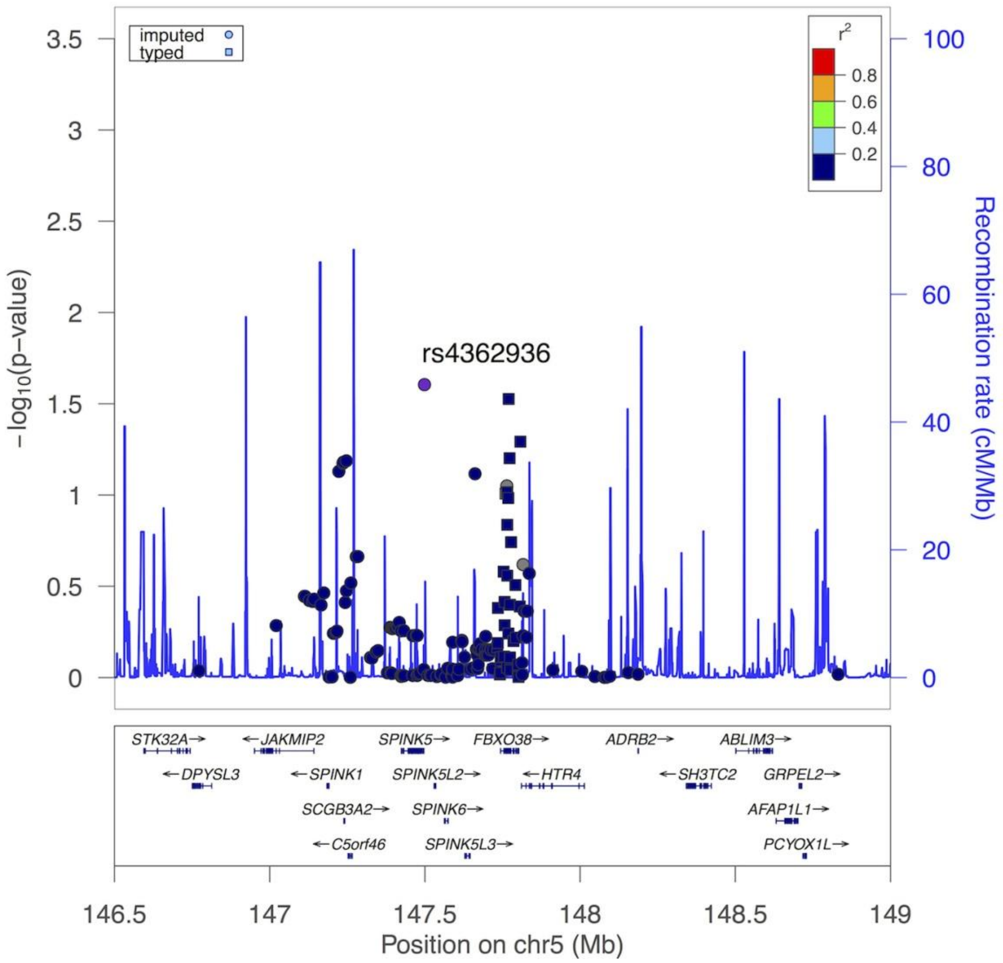

Figure S2. **Regional association plots based on imputed regions on chromosome 5.** Imputed SNPs were indicated as circle and genotyped SNPs were indicated as square. The most significant imputed SNP was chosen as reference SNP (rs4362936).

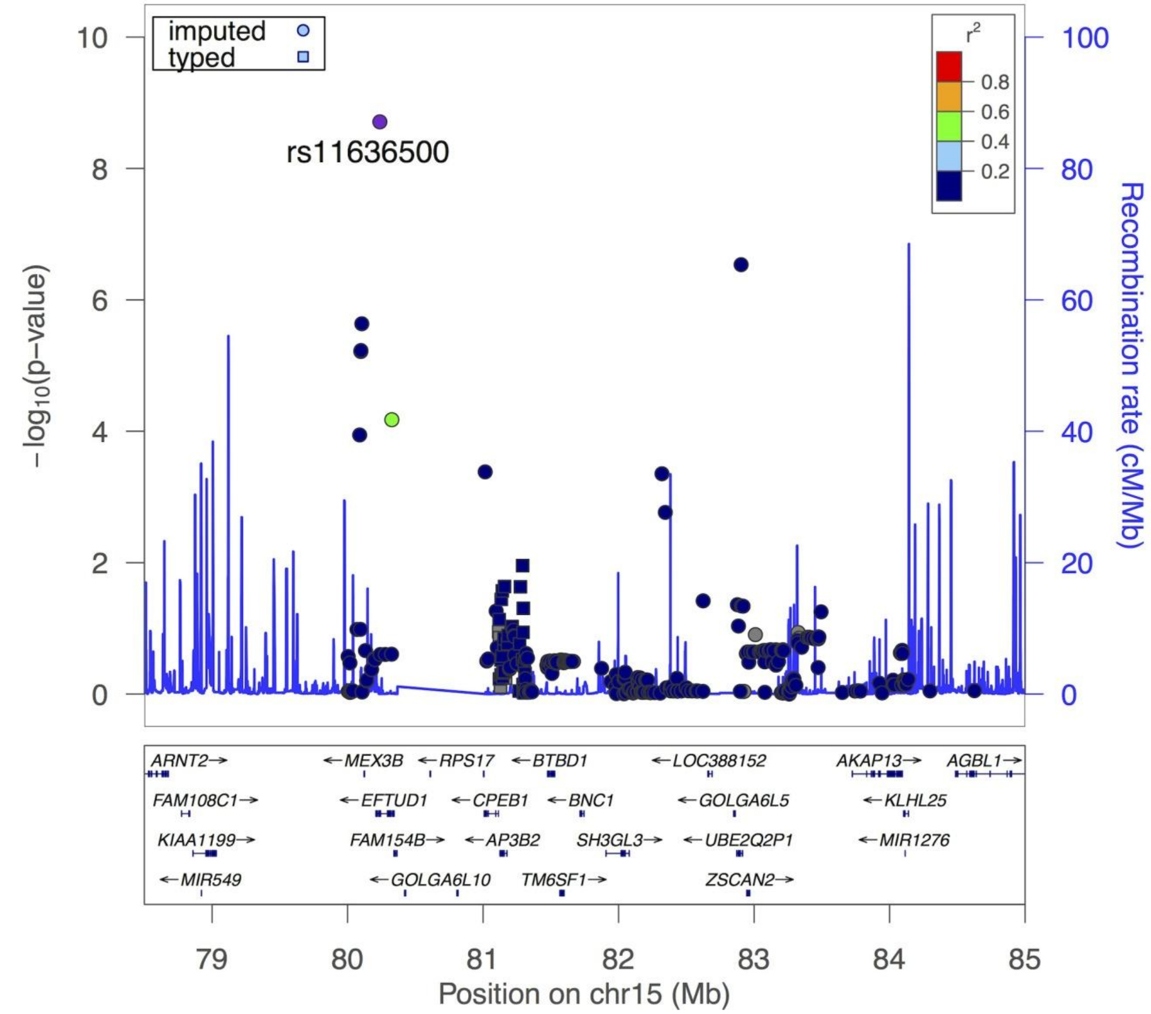

Figure S3. **Regional association plots based on imputed regions on chromosome 15.** Imputed SNPs were indicated as circle and genotyped SNPs were indicated as square. The most significant imputed SNP was chosen as reference SNP (rs1163650).
